# Rapid Fog-Removal Strategies for Traffic Environments

**DOI:** 10.3390/s23177506

**Published:** 2023-08-29

**Authors:** Xinchao Liu, Liang Hong, Yier Lin

**Affiliations:** College of Mechanical Engineering, Tianjin University of Science and Technology, Tianjin 300222, China; liuxinchao@mail.tust.edu.cn (X.L.); hongliang@tust.edu.cn (L.H.)

**Keywords:** fast defogging strategy, dark channel prior, bilinear interpolation, upsampling, Gaussian transform

## Abstract

In a foggy traffic environment, the vision sensor signal of intelligent vehicles will be distorted, the outline of obstacles will become blurred, and the color information in the traffic road will be missing. To solve this problem, four ultra-fast defogging strategies in a traffic environment are proposed for the first time. Through experiments, it is found that the performance of Fast Defogging Strategy 3 is more suitable for fast defogging in a traffic environment. This strategy reduces the original foggy picture by 256 times via bilinear interpolation, and the defogging is processed via the dark channel prior algorithm. Then, the image after fog removal is processed via 4-time upsampling and Gaussian transform. Compared with the original dark channel prior algorithm, the image edge is clearer, and the color information is enhanced. The fast defogging strategy and the original dark channel prior algorithm can reduce the defogging time by 83.93–84.92%. Then, the image after fog removal is inputted into the YOLOv4, YOLOv5, YOLOv6, and YOLOv7 target detection algorithms for detection and verification. It is proven that the image after fog removal can effectively detect vehicles and pedestrians in a complex traffic environment. The experimental results show that the fast defogging strategy is suitable for fast defogging in a traffic environment.

## 1. Introduction

With the continuous improvement in people’s quality of life and travel demands, accompanied by the increase in the number of vehicles year on year, the increase in the total number of vehicles has led to an increase in road safety accidents, especially in a poor traffic environment, such as on foggy days, and the frequency of accidents is increasing. Therefore, it is of great importance to improve the detection of vehicles and pedestrians in foggy traffic environments, and to accurately perceive information regarding vehicles and pedestrians facing the road, in order to reduce the probability of traffic accidents. In a foggy traffic environment, a large number of particles are suspended in the air, which scatter low, and attenuate the reflected low of the target object in the environment; the low directly received by the driver is also affected by the reflected low, resulting in changes in the driver’s observation of the environment color, target proportion, clarity, and other relevant information [1,2,3]. The detailed information is highly blurred [4], resulting in a low visibility on the road, and a limited field of view [5,6], which increases the difficulty in identifying the distance to the front and rear of vehicles and road signs, and in detecting obstacles in the traffic environment in the night haze environment [7,8]. These problems are major challenges for image processing and information retrieval in the later stages. Therefore, it is particularly important for the vision-only autonomous vehicle to perform automatic driving in the foggy traffic environment, and to process the foggy traffic environment information quickly and clearly.

The object detection of vehicles and pedestrians in fog is the only way to achieve autonomous driving and intelligent transport systems. In order to better meet the requirement that automatic driving can detect vehicles and pedestrians on the road in real time under foggy conditions, the detection process is divided into two stages. The first stage is to remove fog from the perceived environment, and the second stage is to detect pedestrians in the traffic environment, based on fog removal. The main innovation in this study is the optimization of the first stage. A fog-removal strategy is proposed that can not only preserve the feature information of the fogged images, but also improve the efficiency of the fogged images. Then, the effectiveness of the defogging strategy is verified through its combination with the existing target detection algorithm.

At present, the mainstream defogging algorithms are mainly divided into three categories. The first category is the image enhancement of image information acquired on foggy days [9], which mainly enhances image details by improving the color, feature contour, and contrast of the image, to make the processed image look clearer. The advantage of this type of algorithm is that the computational process is simple, and the real-time fog-removal result is relatively strong. The disadvantage is that the image processing details are not taken into account too much when the image is defogged, which means that the details of the image information are severely damaged on foggy days after defogging. Among the image enhancement algorithms, the representative algorithms are histogram equalization [10], retinex [11], automatic color enhancement (ACE) [12], etc. The second type aims to recover image information on foggy days [3,13,14]. The image restoration is mainly based on the physical model of atmospheric scattering. Through the observation and summarization of a large number of images with fog and images without fog, the potential mapping relationship is mined, and the inverse calculation is carried out according to the formation process of foggy images, so as to achieve the effect of fog removal. The advantage of this type of algorithm is that the fog-removal effect is good, but the disadvantage is that the real-time performance is relatively poor. Among the defogging algorithms for image restoration, the most representative algorithm is the dark channel prior algorithm [3], which has a good defogging effect. Therefore, a large number of optimization algorithms based on the dark channel prior algorithm have appeared. The third category is the fog-removal algorithm based on deep learning [15,16,17,18,19,20]. The fog-removal algorithm based on deep learning mainly uses a generative adversarial network and convolutional neural network to defog images. The CNN-based method is mainly used to defog the fog image, by estimating the transmission and atmospheric low value in the physical scattering model, or to directly train a large number of defogging data, to realize the transformation of the foggy image into the defogged image. The advantage of this algorithm is that it improves the clarity of foggy images, and enhances the information features of foggy images. The disadvantage is that there will be noise artefacts in the optimized image. It is difficult for the above algorithms to meet the requirements of the accurate and real-time target detection of vehicles and pedestrians by autonomous vehicles in foggy environments. In order to meet the requirements of the accurate and real-time target detection of vehicles and pedestrians in foggy environments, an optimization strategy is proposed that can efficiently remove fog from foggy images, under the premise of retaining image feature information.

In a foggy traffic environment, the detection process is divided into two stages. The first stage is to defog the foggy images by combining the optimization strategy with the defogging algorithm; the second stage is to feed the defogged images into the target detection algorithm for target detection. Target detection algorithms are mainly divided into two categories. The first is the traditional target detection algorithm [21,22]. The traditional target detection algorithm mainly uses a feature extractor to extract image features, and relies on a sliding window to generate candidate regions, which involves complicated calculation methods, a slow detection speed, and a low detection accuracy. Therefore, it is not suitable for application in the target detection process of autonomous vehicles. The second type is the object detection algorithm based on deep learning, and the object detection algorithm based on deep learning is divided into two types. The first type is the candidate region-based object detection algorithm, also known as the two-stage object detection algorithm. The first stage is mainly to distinguish the foreground and background of the image, and the second stage relies on CNN to extract the features of the region of interest for classification and regression. The representative algorithms are R-CNN [23], Mask R-CNN [24], Fast R-CNN [25], Faster R-CNN [26], etc. The second type is the regression-based target detection algorithm. The single-stage target detection algorithm does not need to extract the target candidate region, but directly extracts the information features of the image; this effectively improves the detection speed of the image. The typical algorithms are YOLOv1 [27], YOLOv2 [28], YOLOv3 [29], YOLOv4 [30], YOLOv5 [31], YOLOv6 [32], YOLOv7 [33], SSD series [34,35,36], and so on.

In order to achieve the accurate real-time target detection of vehicles and pedestrians in foggy environment, this paper proposes four optimization strategies to remove fog in foggy images. (1) The first optimization strategy is that the rows and columns of the original images are reduced to 16 times the original size, and the total size of the images is reduced by 256 times after Gaussian transformation, and subjected to 4-time downsampling processing, to improve the processing efficiency for foggy pictures. Then, the fog removal of the picture is conducted for 4-time upsampling, the picture rows and columns are expanded to 16 times the original size, the total size of the picture is 256 times the reduced picture, and then Gaussian transform is used. (2) The second optimization strategy is to reduce the rows and columns of the original picture to 16 times the original size, and 256 times the total size of the picture, through Gaussian transformation and 4-time downsampling processing, to improve the processing efficiency of the foggy picture. Then, bilinear interpolation is carried out on the picture after fog removal, to expand the rows and columns of the picture after fog removal to 16 times the original size. The total size of the image is 256 times that of the reduced image. (3) The third optimization strategy is to reduce the rows and columns of the original picture to 16 times the original size through bilinear interpolation, and reduce the total size of the picture by 256 times, to improve the processing efficiency of the foggy picture. Then, the picture after fog removal is upsampled 4 times, and the rows and columns of the picture are expanded to 16 times the original size, and the total area is 256 times that of the reduced picture. Then, we perform the Gaussian transform. (4) The fourth optimization strategy is to reduce the rows and columns of the original picture to 16 times the original size, and 256 times the total size of the picture, through bilinear interpolation, to improve the processing efficiency of the foggy picture. To verify the effectiveness of the four optimization strategies, automatic color enhancement (ACE), histogram equalization, and dark channel prior are selected in the defogging algorithms of image enhancement and image restoration, respectively. Through verification and comparison, it is found that, under the scenarios and requirements of this experiment, the fusion effect of the dark channel prior defogging algorithm and Optimization Strategy 3 is better. If researchers choose the four optimization strategies proposed in this study to optimize their own algorithms, they need to combine the experimental results according to different needs. Compared with the original dark channel prior algorithm, the edge of the defogged image becomes clearer, and the color information is enhanced. The fast defogging strategy of traffic environments (the fusion of Fast Defogging Strategy 3 and the original dark channel prior algorithm) and the original dark channel prior algorithm can defog images with different concentrations, and the defogging time can be reduced by 83.93–84.92%. In order to further verify the effectiveness of Fast Defogging Strategy 3, the images after defogging were inputted into the commonly used target detection algorithms YOLOv4, YOLOv5, YOLOv6, and YOLOv7 for detection. The experimental results show that, in complex traffic environment, vehicles and pedestrians in foggy images can be effectively detected via the target detection algorithm after a fast defogging strategy, which further verifies the effectiveness of Fast Defogging Strategy 3 (the fusion of Fast Defogging Strategy 3 and the original dark channel prior algorithm).

## 2. Related Work

### 2.1. Four Optimization Strategies

**Figure 1:**
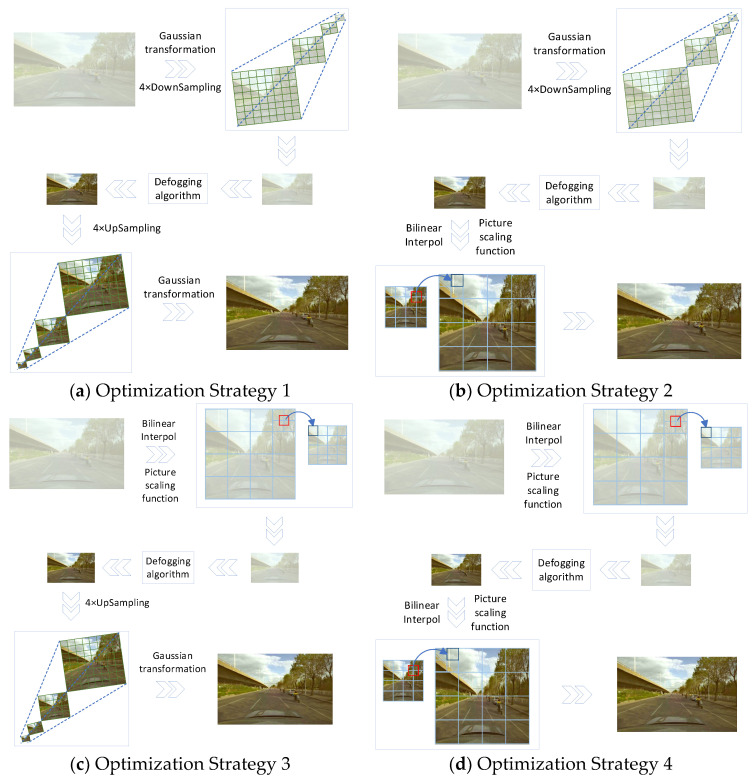
Four defogging strategies. (**a**) Optimisation Strategy 1: the fogged image is first reduced via Gaussian transform and 4-time downsampling processing, to reduce the size of the original image by 256 times, which improves the processing efficiency of the fogged image. Then, the defogging image is upsampled 4 times, and the total size of the image is 256 times that of the reduced image, and then Gaussian transform is performed. (**b**) Optimisation Strategy 2: the foggy image is first reduced via Gaussian transform, and downsampled 4 times, to improve the processing efficiency of the foggy image by 256 times. Then, bilinear interpolation is performed on the fogged image, to enlarge the fogged image by 256 times. (**c**) Optimisation Strategy 3: the foggy image is first reduced by 256 times via bilinear interpolation, to improve the processing efficiency of the foggy image, and then the image after fog removal is up-sampled 4 times, and the total area is 256 times that of the reduced image, and then Gaussian transformation is performed. (**d**) Optimisation Strategy 4: the foggy image is reduced by 256 times via bilinear interpolation, to improve the processing efficiency of the foggy image, and then the image after fog removal is enlarged by 256 times via bilinear interpolation.

At present, the method of detecting obstacles in a foggy environment is mainly the combination of a defogging algorithm and a target detection algorithm. The defogging algorithm will reduce the feature information of the image in the process of defogging, and the reduction in image feature information directly leads to a decrease in the target detection accuracy. Therefore, we need to choose a high-quality defogging algorithm to enhance the characteristics of the target information, but usually the required detection time of the complete defogging algorithm will be substantial. In order to improve the efficiency of fog removal, four strategies are proposed to accelerate fog removal Figure 1.

### 2.2. Gaussian Transform

Gaussian transform [37] is a smoothing and filtering algorithm based on the Gaussian function, which can effectively reduce the noise between pixels, and produce clearer and smoother images. The Gaussian transform is a linear smoothing filter algorithm that mainly weights and sums the pixel information around each pixel in the image, to obtain a new pixel value. The weight of each pixel is determined by the value in the Gaussian convolution kernel function. The Gaussian convolution kernel function is a two-dimensional Gaussian distribution function that is used to calculate the weight of pixels around each pixel point. Through adjustment in the size and standard deviation of the convolution kernel, different degrees of smoothing and noise reduction are produced, as shown in Equation (1) below:

(1) $$I\left(x,y\right)=M\left(x,y\right)\times G\left(x,y\right)=\iint M\left(u,v\right)G\left(x-u,y-v\right)dudv$$

In Formula (1), $M\left(x,y\right)$ is the pixel information of the original image, × represents the convolution operation of $M\left(x,y\right)$ and $G\left(x,y\right)$, $I\left(x,y\right)$ is the smoothed image, and $G\left(x,y\right)$ is the Gaussian kernel function; the specific formula is as follows (2):

(2) $$G\left(x,y\right)=\frac{1}{2\pi \sigma^{2}}e^{-\frac{x^{2}+y^{2}}{2\sigma^{2}}}$$

In Formula (2), σ is the standard deviation of the Gaussian kernel function, which is mainly used to calculate the weight of the pixels around each pixel. As the standard deviation increases, the weight of the pixels around each pixel becomes weaker, and the smoothness of the image becomes more apparent. So, Formula (2) gives a smooth image.

### 2.3. Bilinear Interpolation Algorithm

The bilinear interpolation algorithm [38] mainly calculates the pixel points on the two-dimensional image as the new pixel values. The bilinear interpolation algorithm mainly relies on the four closest pixel values around the pixel to derive the new pixel value, and these four selected pixels must form a rectangular area around the new pixel, as shown in Equation (3):

(3) $$f_{1}=\frac{\left(x_{2}-x\right)f\left(x_{1},y\right)+\left(x-x_{1}\right)f\left(x_{2},y\right)}{x_{2}-x_{1}}$$

In Formula (3), linear interpolation is mainly performed on the two closest pixel points in the horizontal direction, where $\left(x,y\right)$ are the calculated coordinates of the new pixel points, the coordinate values are non-integers in the original image, $\left(x,y_{1}\right)$ and $\left(x,y_{2}\right)$ are the two closest pixel points in the original image, and the values satisfy $y_{1}\leq x\leq y_{2}$. Formula (4) performs a weighted average of $f_{1}$ and $f_{2}$ to obtain the final pixel value, as shown in Formula (4):

(4) $$f\left(x,y\right)=\frac{\left(x_{2}-x\right)\left(y_{2}-y\right)f\left(x_{1},y_{1}\right)+\left(x-x_{1}\right)\left(y_{2}-y\right)f\left(x_{2},y_{1}\right)+\left(x_{2}-x\right)\left(y-y_{1}\right)f\left(x_{1},y_{2}\right)+\left(x-x_{1}\right)\left(y-y_{1}\right)f\left(x_{2},y_{2}\right)}{\left(x_{2}-x_{1}\right)(y_{2}-y_{1})}$$

In Formula (4), $f\left(x_{i},y_{i}\right)$ is the nearest pixel value. Bilinear interpolation involves calculating the value of the new pixel point, by using linear interpolation and weighted averaging in the horizontal and vertical directions. Bilinear interpolation is implemented in combination with the image resize function, as shown in Equation (5), below:

(5) $$T\left(x,y\right)=Y(\frac{x}{fx},\frac{y}{fy})$$

In Formula (5), Y represents the original image, T represents the adjusted image, fx and fy are the scaling factors adjusted according to the required image scale; the smaller the scaling factor in the process of reducing the image $\left(0<fx<1,\;0<fy<1\right)$, the smaller the size of the adjusted image. The larger the scaling factor when enlarging the image $\left(1<fx,\;1<fy\right)$, the larger the size of the adjusted image. x and y are the coordinates of the adjusted image.

## 3. Experiment

In order to verify the effectiveness of the four optimization strategies, ACE, histogram equalization, and dark channel prior algorithm are selected to combine the optimization strategies. In the experiment, high-concentration fog pictures, medium-concentration fog pictures, and low-concentration fog pictures are selected for defogging processing, and the algorithm with a better defogging effect and a stronger real-time performance is identified and analyzed. To better reflect the effectiveness of the defogging strategy, researchers can adjust the size of the defogging pictures according to the requirements of clarity and real-time.

### 3.1. Experimental Environment

In order to optimize the defogging algorithm and test the defogged pictures in complex traffic environment with a deep learning object detection algorithm, the configuration of the experimental environment is shown in Table 1.

### 3.2. Experimental Data

**Figure 2:**
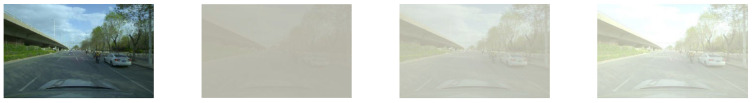
No-fog picture, high-concentration-fog picture, medium-concentration-fog picture, and low-concentration-fog picture.

The foggy picture required in this experiment is the effect created by blending the foggy mask image with the original image. The color of all the pixels in the foggy mask image is set to (R = 166, G = 178, B = 180), resulting in a grey–blue color on the image. The fog concentration is controlled via setting of the weight of the mask image and the original image fusion. The weight range of the mask image and the original image fusion is from 0 to 1, with two decimal places. The closer the weight is to 1, the higher the weight of the original image; the closer the weight is to 0, the higher the weight of the mask image. In this experiment, the weight set for high-concentration fog pictures is a random value ranging from 0.1 to 0.2, the weight set for medium-concentration fog pictures is a random value ranging from 0.25 to 0.35, and the weight set for low-concentration fog pictures is a random value ranging from 0.5 to 0.6, as shown in Figure 2, below. After fog images are obtained in the experiment, deep learning algorithms are required for verification. The dataset adopted by the deep learning model is BDD100K [39], which contains pictures in foggy, cloudy, rainy, snowy, day, night, and other traffic environments. A total of 10,000 pictures including pedestrians and vehicles are selected from BDD100K as the training set for this experiment. The validation set and the test set, respectively, adopt 1000 self-made datasets, as shown in Figure 2.

### 3.3. Analysis of Experimental Results

To allow for better observation and analysis of the processed pictures, a fog-free picture collected by the data is shown in Figure 3. ACE, histogram equalization, and the dark channel prior algorithm combined four optimization strategies, to defog pictures with a high concentration, medium concentration, and low concentration, respectively. The defogging effect is shown in Figure 4, Figure 5, Figure 6, Figure 7, Figure 8, Figure 9, Figure 10, Figure 11, Figure 12, Figure 13, Figure 14 and Figure 15.

**Figure 4:**
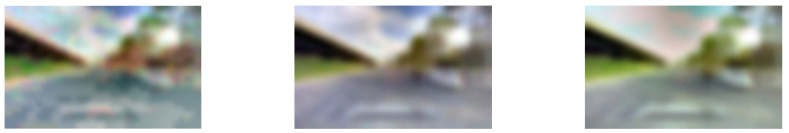
The combination of the ACE algorithm and Strategy 1. After Gaussian transformation and 4-time downsampling processing, the size of the original picture is reduced by 256 times, which improves the processing efficiency of the foggy picture. After the ACE algorithm is defogged, the picture after defogging is upsampled by 4 times. The image is enlarged to 256 times the original size, and then the Gaussian transform is performed, to obtain the image.

**Figure 5:**
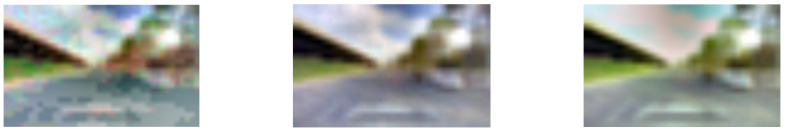
The combination of the ACE algorithm and Strategy 2. Images with high-concentration fog, medium-concentration fog, and low-concentration fog are reduced by 256 times after Gaussian transformation and 4-time downsampling processing, which improves the processing efficiency for foggy images. After defogging via the ACE algorithm, the picture is obtained through the use of bilinear interpolation, to enlarge the picture 256 times after the fog removal.

**Figure 6:**
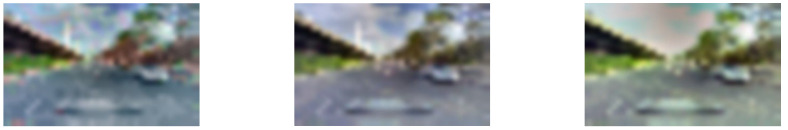
The combination of the ACE algorithm and Strategy 3. Images with a high concentration, medium concentration, and low concentration of fog are reduced by 256 times via bilinear interpolation, to improve the processing efficiency for foggy images. After defogging via the ACE algorithm, the defogging images are upsampled 4 times, and the images are enlarged to 256 times the original size. Then, the Gaussian transform produces the picture.

**Figure 7:**
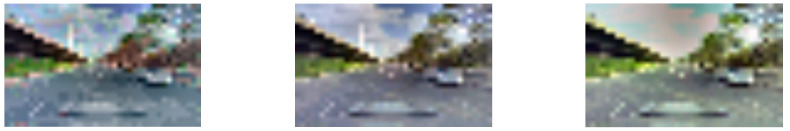
The combination of the ACE algorithm and Strategy 4. High-fog, medium-fog, and low-fog images are reduced by 256 times via bilinear interpolation, to improve the processing efficiency for foggy images. After defogging via the ACE algorithm, images with defogging are reconstructed via bilinear interpolation, and images with defogging are enlarged by 256 times.

Figure 4, Figure 5, Figure 6 and Figure 7 are the defogging images obtained via the ACE algorithm, combined with the four optimization strategies (a–d) in Figure 1. The corresponding optimization strategy in Figure 4 is that the foggy images are defogged via the ACE algorithm, after undergoing Gaussian transformation and 4-time downsampling processing, and then the defogging images are defogged via 4-time upsampling and Gaussian transformation. Comparing Figure 4 with Figure 5, Figure 6 and Figure 7, it can be seen that Defogging Strategy 1, corresponding to Figure 4, has a better noise removal ability than the Defogging Strategy 2, Strategy 3, and Strategy 4 in Figure 5, Figure 6 and Figure 7, and the ambiguity in Figure 4 is higher than that in Figure 5, Figure 6 and Figure 7. Figure 5 shows that the foggy picture is defogged by the ACE algorithm after Gaussian transformation and 4-time downsampling processing, and then the defogged picture is defogged via bilinear interpolation and image enlargement. Comparing Figure 5 with Figure 4, Figure 6 and Figure 7, it can be seen that Defogging Strategy 2, in Figure 5, shows a better noise removal ability than Defogging Strategy 3 and Strategy 4, in Figure 6 and Figure 7, while Defogging Strategy 2, in Figure 5, has a worse noise removal ability than Defogging Strategy 1, in Figure 4. The fuzziness of Figure 5 is lower than that of Figure 4, and higher than that of Figure 6 and Figure 7. Figure 6 shows the bilinear interpolation of, and reduction in, the fogged picture, defogging via the ACE algorithm, and then the 4-fold upsampling and Gaussian transformation of the defogging picture. Comparing Figure 6 with Figure 4, Figure 5 and Figure 7, it can be seen that Defogging Strategy 3, in Figure 6, shows a better noise removal capability than Defogging Strategy 4, in Figure 7, while Defogging Strategy 3, in Figure 6, has a worse noise removal capability than Defogging Strategy 1 and Defogging Strategy 2, in Figure 4 and Figure 5. The vagueness of Figure 6 is lower than that of Figure 4, and that of Figure 5 is higher than that of Figure 7. Figure 7 shows the bilinear interpolation of, and reduction in, foggy pictures, defogging via the ACE algorithm, and then the bilinear interpolation and image enlargement of defogged pictures. Comparing Figure 7 with Figure 4, Figure 5 and Figure 6, it can be seen that Defogging Strategy 4, corresponding to Figure 7, has a poor ability to remove noise compared with Defogging Strategy 1, Strategy 2, and Strategy 3, of Figure 4, Figure 5 and Figure 6, and the ambiguity of Figure 7 is lower than that of Figure 4, Figure 5 and Figure 6. Based on the fuzzy degree of the image, and the ability of the defogging algorithm to remove noise, it is found that the vehicle features in the defogging pictures in Figure 5 and Figure 6 are clearer than those in Figure 4, and the defogging pictures in Figure 5 and Figure 6 have less noise than those in Figure 7. Optimization Strategy 2, corresponding to Figure 5, and Optimization Strategy 3, corresponding to Figure 6, perform better among the four optimization strategies.

**Figure 8:**
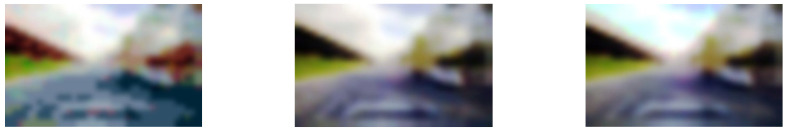
The combination of the histogram equalization algorithm and Strategy 1. The size of the original picture is reduced by 256 times via Gaussian transformation, and 4 times via downsampling, which improves the processing efficiency of the foggy picture. After histogram equalization and fog removal, the picture after fog removal is upsampled by 4 times. The image is enlarged to 256 times the original size, and then the image is obtained via Gaussian transformation.

**Figure 9:**
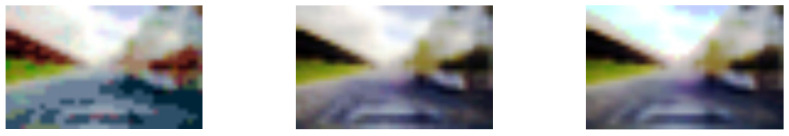
The combination of the histogram equalization algorithm and Strategy 2. Images with a high concentration of fog, medium concentration of fog, and low concentration of fog are reduced by 256 times after Gaussian transformation and 4-time downsampling processing, which improves the processing efficiency for foggy images. After histogram equalization to remove fog, the image with fog removed is enlarged 256 times via bilinear interpolation.

**Figure 10:**
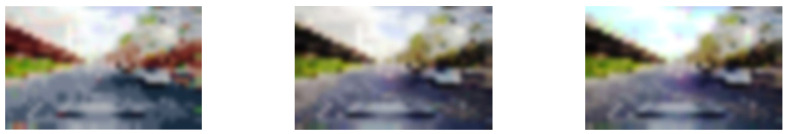
The combination of the histogram equalization algorithm and Strategy 3. Images with high concentration of fog, medium concentration of fog, and low concentration of fog are reduced by 256 times via bilinear interpolation, to improve the processing efficiency for foggy images. After histogram equalization and fog removal, the image with fog removed is upsampled 4 times, and the image is enlarged to 256 times the original size. Then, the Gaussian transform is applied to the picture.

**Figure 11:**
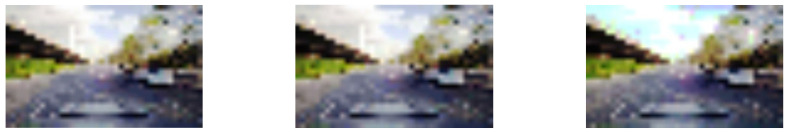
The combination of the histogram equalization algorithm and Strategy 4. The images with a high concentration of fog, medium concentration of fog, and low concentration of fog are reduced by 256 times via bilinear interpolation, to improve the processing efficiency for foggy images. After histogram equalization to remove fog, the image with fog removed is enlarged 256 times via bilinear interpolation.

Figure 8, Figure 9, Figure 10 and Figure 11 are the defogging images obtained through combining the histogram equalization algorithm with the four optimization strategies a–d in Figure 1. The corresponding optimization strategy in Figure 8 is to defog the foggy images via the histogram equalization algorithm, after Gaussian transformation and 4-time downsampling processing. Then, the image after fog removal is up-sampled 4 times, and undergoes Gaussian transform. Comparing Figure 8 with Figure 9, Figure 10 and Figure 11, it can be seen that the Defog Strategy 1, corresponding to Figure 8, has a better noise removal ability than Defog Strategy 2, Strategy 3, and Strategy 4, of Figure 9, Figure 10 and Figure 11, and the ambiguity of Figure 8 is higher than that of Figure 9, Figure 10 and Figure 11. Figure 9 shows that the foggy picture is defogged via the histogram equalization algorithm, after Gaussian transformation and 4-time downsampling processing, and then the defogged picture is defogged via bilinear interpolation and image enlargement. Comparing Figure 9 with Figure 8, Figure 10 and Figure 11, it can be seen that Defogging Strategy 2, in Figure 9, shows a better noise removal ability than Defogging Strategy 3 and Strategy 4, in Figure 10 and Figure 11, while Defogging Strategy 2, in Figure 9, has a worse noise removal ability than Defogging Strategy 1, in Figure 8. The vagueness of Figure 9 is lower than that of Figure 8, and higher than that of Figure 10 and Figure 11. Figure 10 shows the bilinear interpolation of, and reduction in, the foggy picture, defogging via the histogram equalization algorithm, and then the 4-fold upsampling and Gaussian transformation of the defogging picture. Comparing Figure 10 with Figure 8, Figure 9 and Figure 11, it can be seen that Defogging Strategy 3, in Figure 10, has a better noise removal ability than Defogging Strategy 4, in Figure 11, while Defogging Strategy 3, in Figure 10, has a worse noise removal ability than Defogging Strategy 1 and Strategy 2, in Figure 8 and Figure 9. The vagueness of Figure 10 is lower than that of Figure 8, and that of Figure 9 is higher than that of Figure 11. Figure 11 shows the bilinear interpolation of, and reduction in, foggy pictures, defogging via the histogram equalization algorithm, and then the bilinear interpolation and image enlargement of defogging pictures. Comparing Figure 11 with Figure 8, Figure 9 and Figure 10, it can be seen that Defogging Strategy 4, corresponding to Figure 11, shows a poor noise removal ability compared with Defogging Strategy 1, Strategy 2, and Strategy 3, of Figure 8, Figure 9 and Figure 10, and the ambiguity of Figure 11 is lower than that of Figure 8, Figure 9 and Figure 10. Based on the fuzzy degree of the image, and the ability of the defogging algorithm to remove noise, it is found that the vehicle features in the defogging pictures in Figure 9 and Figure 10 are clearer than those in Figure 8; the white blur at the junction between the edge of the viaduct and the sky in the defogging pictures in Figure 10 is also lesser than that in Figure 8 and Figure 9; the noise in Figure 9 and Figure 10 is lesser than that in Figure 11. Among the four optimization strategies, Defog Strategy 2, corresponding to Figure 9, and Defog Strategy 3, corresponding to Figure 10, perform better.

**Figure 12:**
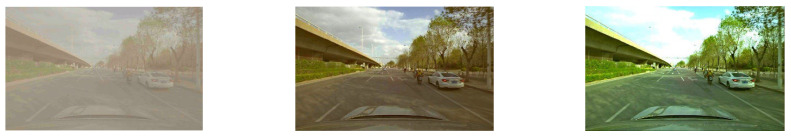
The dark channel prior algorithm combined with Strategy 1. After Gaussian transformation and 4-time downsampling, the size of the original picture is reduced in size by 256 times, which improves the processing efficiency of the fogged picture. After the dark channel is defogged, the defogged picture is upsampled by 4 times. The image is enlarged to 256 times the original size, and then the image is obtained via Gaussian transformation.

**Figure 13:**
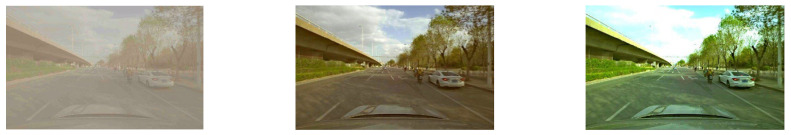
The combination of the dark channel prior algorithm and Strategy 2. Images with a high concentration, medium concentration, and low concentration of fog are reduced by 256 times after Gaussian transformation and 4-time downsampling processing, which improves the processing efficiency for foggy images. The picture is obtained via bilinear interpolation, to enlarge the picture 256 times after the fog removal.

**Figure 14:**
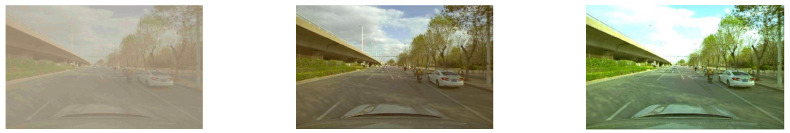
The combination of the dark channel prior algorithm and Strategy 3. Images with a high concentration, medium concentration, and low concentration are reduced by 256 times via bilinear interpolation, to improve the processing efficiency for fogged images. After dark channel defogging, the images with fog removed are upsampled 4 times, and the images are expanded to 256 times the original size. Gaussian transformation is then applied to the images.

**Figure 15:**
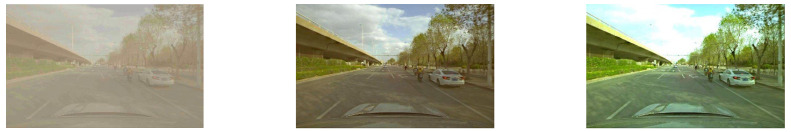
The combination of the dark channel prior algorithm and Strategy 4. Images with a high concentration, medium concentration, and low concentration of fog are reduced by 256 times through bilinear interpolation, to improve the processing efficiency for foggy images. After the dark channel is defogged, images with defogging are reconstructed via bilinear interpolation, and the defogged images are enlarged by 256 times.

Figure 12, Figure 13, Figure 14 and Figure 15 are the defog images obtained via the dark channel prior algorithm combined with the four optimization strategies a–d in Figure 1. The defog images in Figure 12, Figure 13, Figure 14 and Figure 15 show no difference from the visualized images. The comparison between Figure 4 and Figure 5 and Figure 8 and Figure 9 shows that the trees next to the road in Figure 8 and Figure 9 are transformed into white fog, and the white boundary range at the junction between the edge of the viaduct and the sky will increase with the increase in the concentration of the foggy pictures. The comparison between Figure 6 and Figure 7 and Figure 10 and Figure 11 shows that the clarity of the vehicle features in Figure 6 and Figure 7 is higher than that in Figure 10 and Figure 11. Through comparing the defogging pictures from Figure 4 to Figure 11, it is found that different defogging algorithms and different defogging strategies will produce different defogging effects. Through a comparison of the defogging images from Figure 4 to Figure 15, it is found that the dark channel prior defogging algorithm is more suited to being combined with the four optimization strategies a–d in Figure 1, and shows a better defogging effect compared with ACE and histogram equalization.

**Figure 16:**
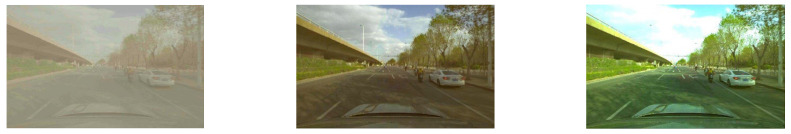
The pictures with high-concentration fog, medium-concentration fog, and low-concentration fog, after the original dark channel defogging algorithm.

**Figure 17:**
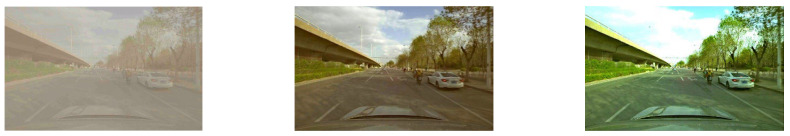
The pictures of high-concentration fog, medium-concentration fog, and low-concentration fog, after Optimization Strategy 2 (referring to the fusion of dark channel prior and Optimization Strategy 2).

**Figure 18:**
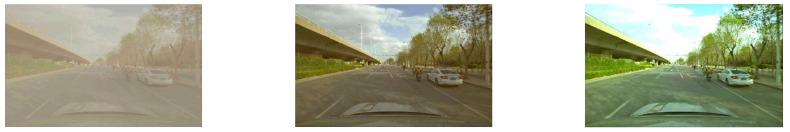
The pictures with high-concentration fog, medium-concentration fog, and low-concentration fog after Optimization Strategy 3 (referring to the fusion of dark channel prior and Optimization Strategy 3).

From the comparison of the above experimental results, we can find that the performance of optimization Strategies 2 and 3 in Figure 1 is better than that of optimization Strategies 1 and 4 in Figure 1. In the experiment, the dark channel prior defogging algorithm is selected to be combined with Optimization Strategy 2 and Optimization Strategy 3, to optimize, and compare with, the original dark channel prior defogging algorithm. Figure 16, Figure 17 and Figure 18 were obtained using defogging pictures with a high concentration, medium concentration, and low concentration, respectively. Combined with the canny [40] algorithm and three-dimensional color distribution, the effectiveness of the original dark channel prior defogging algorithm and Optimization Strategy 2 and Optimization Strategy 3, in Figure 1, was judged.

**Figure 19:**
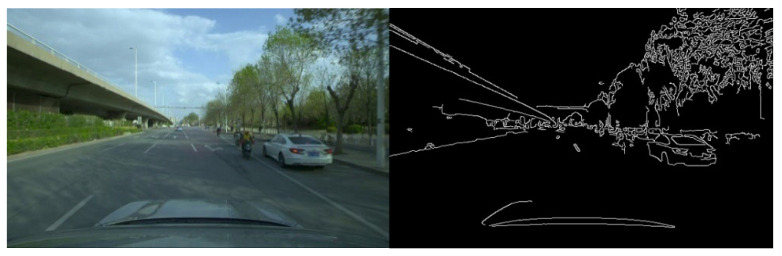
The results obtained from the processing of the fog-free image and the original image via the canny algorithm.

**Figure 20:**
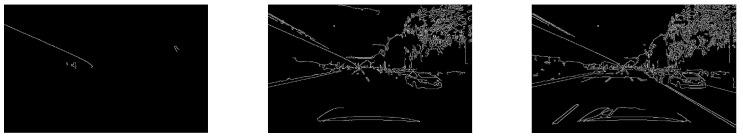
The results obtained via the canny algorithm on the defogging pictures in high-concentration fog, defogging pictures in medium-concentration fog, and defogging pictures in low-concentration fog, obtained via the original dark channel prior defogging algorithm.

Figure 16, Figure 17 and Figure 18 show the original dark channel prior defogging algorithm, the dark channel prior defogging algorithm combined with Optimization Strategy 2 of (b) in Figure 1, and the dark channel prior defogging algorithm combined with Optimization Strategy 3 of (c) in Figure 1, for defogging images of a high concentration, medium concentration, and low concentration. Comparing the visualized fog-removal images in Figure 16, Figure 17 and Figure 18, no difference can be observed with the naked eye. In the experimental analysis, the canny algorithm and three-dimensional color distribution were selected to analyze the performance of the original dark channel prior defogging algorithm, the dark channel prior defogging algorithm combined with Optimization Strategy 2 (b) in Figure 1, and the dark channel prior defogging algorithm combined with Optimization Strategy 3 (c) in Figure 1. In order to better analyze the performance of the optimization strategy, the canny algorithm is used to process the original no-fog picture, as shown in Figure 19. Through comparing the contour line features in Figure 19 with those in Figure 20, it can be seen that the contour line features in the fog-removal picture with a high concentration are lower than those in Figure 19, and there is no obvious difference between the contour line features in the fog-removal picture with a medium concentration in Figure 20, and those in Figure 19. The contour features of the lane lines in the defogging picture with low-concentration fog in Figure 20 are obviously richer and clearer than those in Figure 19. Therefore, we can conclude that the dark channel prior defogging algorithm itself has the performance of enhancing the contour features of objects in the image.

**Figure 21:**
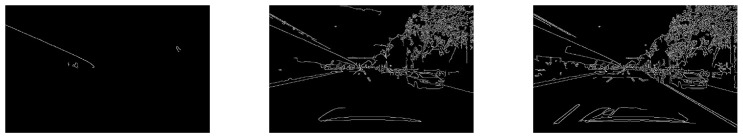
The results obtained via the canny algorithm for the defogging picture for a high-concentration fog day, the defogging picture for a medium-concentration fog day, and the defogging picture for a low-concentration fog day, obtained via Optimization Strategy 2 (referring to the fusion of dark channel prior and Optimization Strategy 2).

**Figure 22:**
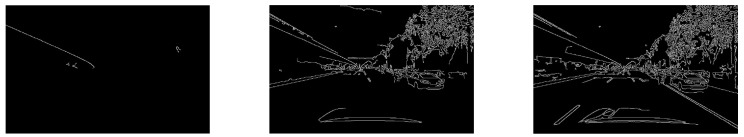
The results obtained via the canny algorithm for the defogging picture for a high-concentration fog day, the defogging picture for a medium-concentration fog day, and the defogging picture for a low-concentration fog day, obtained via Optimization Strategy 3 (referring to the fusion of dark channel prior and Optimization Strategy 3).

We compared Figure 20, Figure 21 and Figure 22, and found that there was basically no difference in the feature contour information among the high-concentration-fog defogging pictures in Figure 20, Figure 21 and Figure 22. The contour features of motorcycles and motorcycle drivers in the fog-removal pictures of a medium concentration in Figure 22 are richer and clearer than those in the fog-removal pictures of a medium concentration in Figure 20 and Figure 21. The outline features of the car in the low-concentration fog-removal pictures in Figure 22 are richer and clearer than those in the low-concentration fog-removal pictures in Figure 20 and Figure 21. We can conclude that the feature contours of the defogging pictures in Optimization Strategy Three (referring to the fusion of dark channel prior and Optimization Strategy 3) are clearer than those in Optimization Strategy Two (referring to the fusion of dark channel prior and Optimization Strategy 2) and the original dark channel prior defogging pictures.

**Figure 23:**
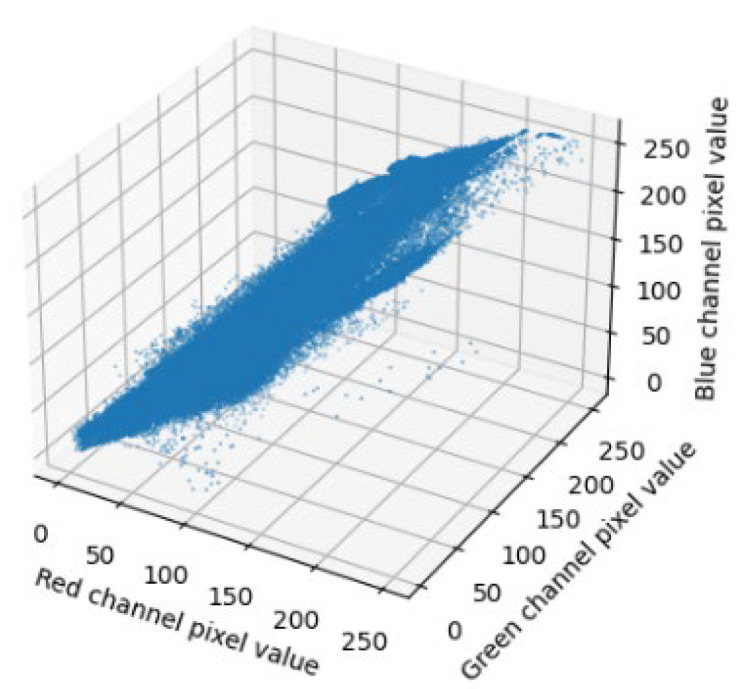
The 3D color distribution in the fog-free image.

**Figure 24:**
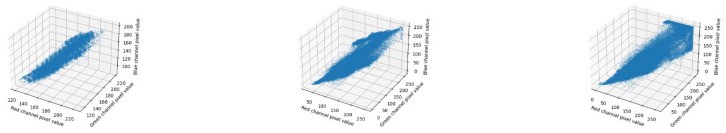
The three-dimensional color distribution in pictures with high-concentration fog, medium-concentration fog, and low-concentration fog, after the original dark channel defogging algorithm.

**Figure 25:**
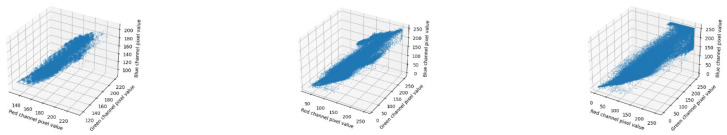
The three-dimensional color distribution in high-concentration, medium-concentration, and low-concentration fog images obtained via optimizing Strategy Two (referring to the fusion of dark channel prior and Optimization Strategy 2).

**Figure 26:**
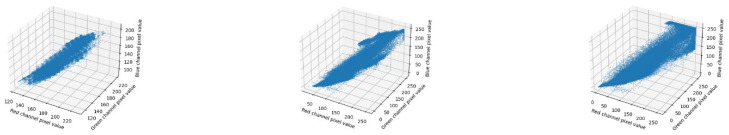
The three-dimensional color distribution in high-concentration, medium-concentration, and low-concentration fog images obtained via Optimizing Strategy Three (referring to the fusion of dark channel prior and Optimization Strategy 3).

In order to further analyze the effectiveness of the optimization strategy, three-dimensional color distribution is carried out in Figure 16, Figure 17 and Figure 18, three-dimensional color distribution of the fog free image is shown in Figure 23, and there is little difference in the three-dimensional color distribution of the fog removal image with high concentration of fog in Figure 24, Figure 25 and Figure 26. Through comparing the three-dimensional color distribution in the fog-removing pictures of medium concentration in Figure 23 with that in the fog-free pictures in Figure 24, Figure 25 and Figure 26, it can be seen that the three-dimensional color distribution in the fog-removing pictures of medium concentration in Figure 25 and Figure 26 is more similar to that in the fog-free pictures in Figure 23, with more balanced pixels. On the other hand, the three-dimensional color distribution diagram of the fog-removing picture in the medium-concentration foggy day in Figure 24 is more dispersed. Through the comparison of the three-dimensional color distribution in the images of low-concentration fog removal in Figure 24, Figure 25 and Figure 26, it can be seen that the three-dimensional color distribution in the images of low-concentration fog removal in Figure 25 and Figure 26 shows a smaller range, and more balanced pixels, while the three-dimensional color distribution in the images of low-concentration fog removal in Figure 24 shows a larger range, and more sparse pixels. It can be concluded that the 3D color distribution in the defogging pictures in Optimization Strategy Three (referring to the fusion of dark channel prior and Optimization Strategy 3) is more balanced than that in optimization Strategy Two (referring to the fusion of dark channel prior and Optimization Strategy 2), and the original dark channel prior defogging pictures.

**Figure 27:**
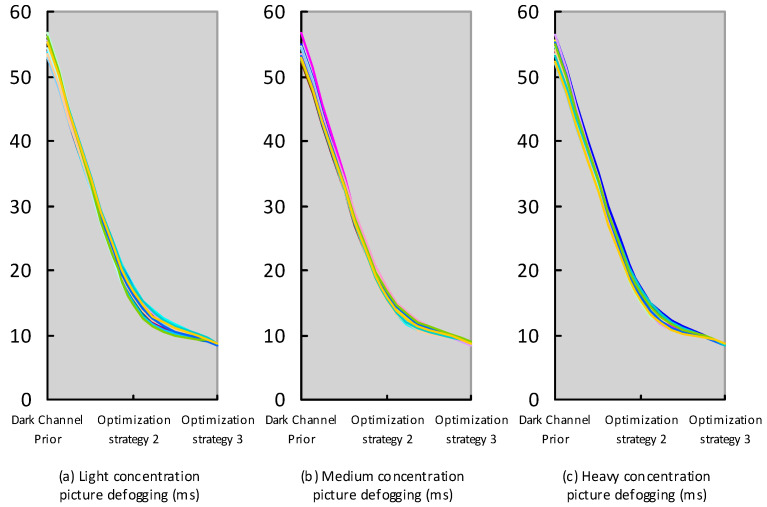
The original dark channel prior algorithm, Optimization Strategy 2 (referring to the fusion of dark channel prior and Optimization Strategy 2), and Optimization Strategy 3 (referring to the fusion of dark channel prior and Optimization Strategy 3). The defogging time curve (**a**–**c**) obtained via 20 groups of defogging experiments on low-concentration fog pictures, medium-concentration fog pictures, and heavy-concentration fog pictures. The vertical axis represents the time taken to defog the image in milliseconds, and the horizontal axis represents the corresponding defog strategy.

In order to further analyze which out of Optimization Strategy 2 and optimization Strategy 3 has a better real-time performance, the optimization strategy with the best real-time performance is compared with the original dark channel prior algorithm. Optimization Strategy 2, Optimization Strategy 3, and the original channel prior algorithm were used to conduct 20 groups of defogging experiments on high-concentration foggy pictures, medium-concentration foggy pictures, and low-concentration foggy pictures, respectively. The real-time defogging performance of Optimization Strategy 2, Optimization Strategy 3, and the original dark channel prior algorithm was compared and analyzed. Figure 27 and Table 2, below, show this.

Figure 27 shows the original dark channel prior algorithm, Optimization Strategy 2 (referring to the fusion of dark channel prior and Optimization Strategy 2), and Optimization Strategy 3 (referring to the fusion of dark channel prior and Optimization Strategy 3). We can see the defogging time curve obtained via 20 groups of defogging experiments on low-concentration fog pictures, medium-concentration fog pictures, and heavy-concentration fog pictures. From the defogging time curve in Figure 27, we can easily see that, compared with dark channel prior and Optimization Strategy 2, Optimization Strategy 3 has the best real-time performance for fogging images with a high concentration, medium concentration, or low concentration. The 20 groups of experiments are listed in Table 2, by dark channel prior, Optimization Strategy 2, and Optimization Strategy 3, respectively. We see the minimum, maximum, and average defogging time of low-concentration fog pictures, medium-concentration fog pictures, and heavy-concentration fog pictures. From Table 2, we can see that the real-time performance of Optimization Strategy 3 is better than the original dark channel prior algorithm and optimization, and whether it is for the minimum, maximum, or average defogging time of low-concentration fog pictures, medium-concentration fog pictures, or heavy-concentration fog pictures, Strategy 2 has a better real-time performance. Optimization Strategy 3 defogs the low-concentration foggy images with the original dark channel prior. Compared with the original dark channel prior, Optimization Strategy 3 reduced the minimum defogging time of low-concentration foggy pictures by 84.14%, the maximum defogging time of low-concentration foggy pictures by 84.92%, and the average defogging time of low-concentration foggy pictures by 84.27%. Optimization Strategy 3 defogged images with a medium concentration with the original dark channel prior. Compared with the original dark channel prior, Optimization Strategy 3 reduced the minimum defogging time of images with a medium concentration by 83.93%, the maximum defogging time of images with a medium concentration by 84.23%, and the average defogging time of images with a medium concentration by 83.93%. Optimization Strategy 3 defogged images with a medium concentration with the original dark channel prior. Compared with the original dark channel prior, Optimization Strategy 3 reduced the minimum defogging time of heavy-concentration foggy pictures by 83.96%, the maximum defogging time of heavy-concentration foggy pictures by 84.56%, and the average defogging time of heavy-concentration foggy pictures by 84.31%. According to comparative analysis, we can conclude that Optimization Strategy 3 has a better real-time performance than the dark channel prior algorithm and Optimization Strategy 2 and, compared with the dark channel prior algorithm, Optimization Strategy 3 has greatly improved the real-time performance and defogging effect.

In order to further verify the effectiveness and advancement of Optimization Strategy 3 (the fusion of dark channel prior and Optimization Strategy 3), the performance of our optimized defog algorithm was compared with that of DCP [3], DCPDN [41], AOD-NET [18], CAP [42], EN-DCP [43] defog algorithms. We obtain the similarity index measure (SSIM) [44], and the peak signal-to-noise ratio (PSNR) [45], in order to calculate the average gradient of a foggy picture. We first convert the color image to a grayscale image, then use Sobel [46] to calculate the gradient on the X and Y axes of the image, then use the Euclidian distance [47] formula to calculate the gradient amplitude and, finally, calculate the average gradient amplitude, to obtain the average gradient. The higher the average gradient, the better the image quality. The SSIM represents the structural similarity index measurement between the original foggy image and the image after fog removal. The higher the value, the closer the image structure after fog removal is to that of the original image. The PSNR is used to evaluate the degree of image distortion, and the non-reference metric of the average gradient is used to measure the fog image information richness. The larger the value of the two types of information, the richer the image information will be, and the clearer the details in the image will be. Table 3, Table 4 and Table 5, below, show this.

Compare the DCP, DCPDN, AOD-NET, and EN-DCP defogging algorithms in Table 3, Table 4 and Table 5 with the SSIM, PSNR, average gradient, and the evaluation indexes of the defogging time of our fast defogging Optimization Strategy 3 (the fusion of dark channel prior and Optimization Strategy 3). It can be seen that our fast fog-removal algorithm (the fusion of dark channel prior and Optimization Strategy 3) shows an excellent detection performance in SSIM, PSNR, average gradient, and fog-removal time. Especially in terms of the defogging time, the fast defogging algorithm (the fusion of dark channel prior and Optimization Strategy 3) takes 84.18% less time to defog images with a light concentration than the original dark channel prior algorithm. The fast defogging algorithm (the fusion of dark channel prior and Optimization Strategy 3) takes 84.15% less time to defog images with a medium concentration than the original dark channel prior algorithm. The fast defogging algorithm (the fusion of dark channel prior and Optimization Strategy 3) takes 84.02% less time to defog images in light-concentration fog than the original dark channel prior algorithm. The comparison results prove the effectiveness of our fast fog-removal optimization Strategy 3 (the fusion of dark channel prior and Optimization Strategy 3).

**Figure 28:**
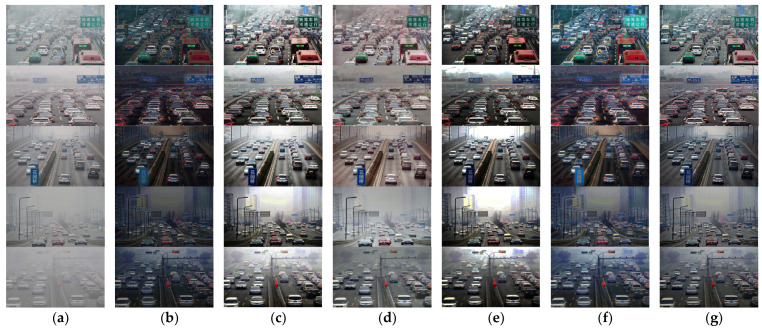
A qualitative comparison of real-world images using different methods. (**a**) The input fog image; (**b**) defogging images obtained via the DCP defogging algorithm; (**c**) defogging images obtained via the DCPDN defogging algorithm; (**d**) defogging images obtained via the AOD-NET defogging algorithm; (**e**) defogging images obtained via the CAP defogging algorithm; (**f**) defogging images obtained via the EN-DCP defogging algorithm; (**g**) defogging images obtained via our defogging algorithm.

In order to further verify the effectiveness of our defogging algorithm on traffic scene defogging, the traffic environment in the real world is selected for defogging treatment, and the comparison is made with the classical defogging algorithm, and the recently developed defogging algorithm, as shown in Figure 28:

Figure 28 shows the qualitative comparison with the results of five advanced defogging algorithms, DCP, DCPDN, AOD-NET, CAP, and EN-DCP in a real foggy traffic environment. Through comparing the result (g) of our defogging algorithm with the result (b) of DCP’s defogging algorithm, it can be easily found that the result picture (g) has a more moderate brightness than that of the result picture (b), and has a better defogging effect on the distant traffic scene, and a clearer outline. Through comparing the result (g) of our defogging algorithm with the result (c) of DCPDN’s defogging algorithm, it can be clearly found that the defogging effect of the result (c) on the distant traffic scene in the image is relatively poor, and there will be ambiguity after defogging. Via comparing the result (g) of our defogging algorithm with the result (d) of AOD-NET’s defogging algorithm, it can be easily found that the contour of the result picture (g) is clearer than that of the result picture (d), and the effect of defogging in the distance is better. Through comparing the result (g) obtained via our defogging algorithm with the result (e) obtained via CAP’s defog algorithm, it can be clearly found that the brightness of the result picture (g) is more moderate than that of the result picture (e), and the result picture (e) obtained via CAP’s defogging algorithm is too bright in the far sky, and too dark in the near traffic scene environment, and the outline is not clear. Through comparison of the result (g) obtained via our defogging algorithm with the result (f) obtained via the defogging algorithm of EN-DCP, it can be clearly found that the contour of the result image (g) is clearer than that of the result image (e), and the result image contour obtained via the defogging algorithm of EN-DCP has artifacts, and the far and near traffic scenes are darker. Comparison with these five algorithms further confirms the outstanding performance of our defogging algorithm in foggy traffic scenarios.

### 3.4. Target Detection and Verification of Fast Fog-Removal Optimization Strategy Three

**Figure 29:**
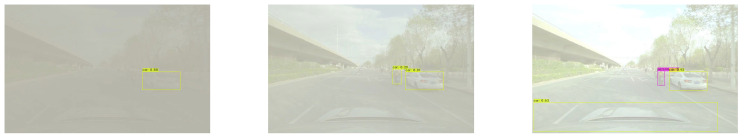
YOLOv4 conducted the detection of vehicles and pedestrians in images with a high concentration, medium concentration, and low concentration, respectively.

**Figure 30:**
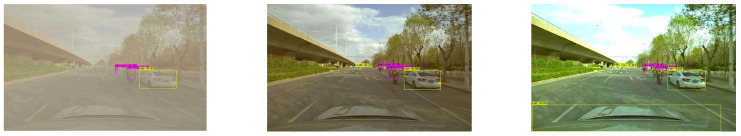
YOLOv4, respectively, detects vehicles and pedestrians in defogging pictures with high-concentration fog, medium-concentration fog, and low-concentration fog.

**Figure 31:**
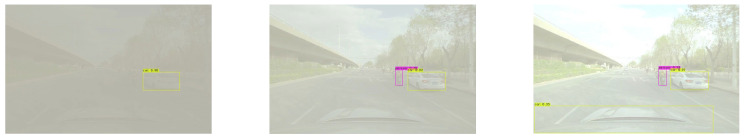
YOLOv5 conducted the detection of vehicles and pedestrians in pictures with a high concentration, medium concentration, and low concentration, respectively.

**Figure 32:**
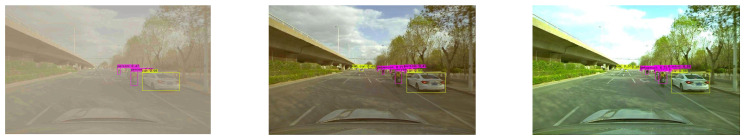
YOLOv5, respectively, detects vehicles and pedestrians in defogging pictures with high-concentration fog, medium-concentration fog, and low-concentration fog.

**Figure 33:**
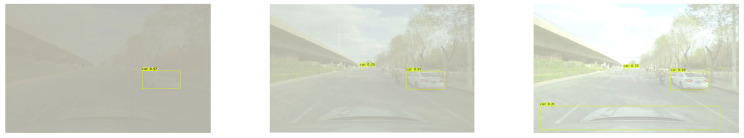
YOLOv6 conducted the detection of vehicles and pedestrians in pictures with a high concentration, medium concentration, and low concentration, respectively.

**Figure 34:**
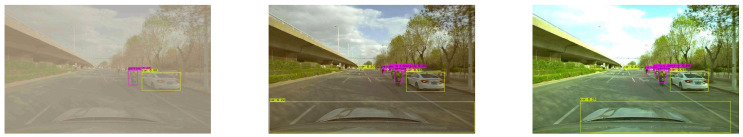
YOLOv6, respectively, detects vehicles and pedestrians in defogging pictures with high-concentration fog, medium-concentration fog, and low-concentration fog.

**Figure 35:**
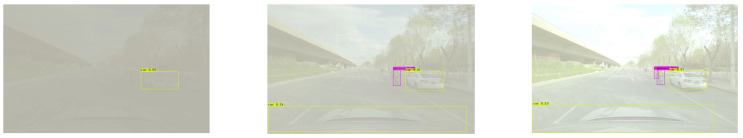
YOLOv7 conducted the detection of vehicles and pedestrians in pictures with a high concentration, medium concentration, and low concentration, respectively.

**Figure 36:**
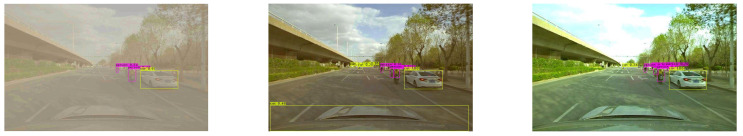
YOLOv7, respectively, detects vehicles and pedestrians in defogging pictures with high-concentration fog, medium-concentration fog, and low-concentration fog.

In order to verify whether the object-detection algorithm can detect the vehicles and pedestrians in the fog-removal image via Optimization Strategy 3, the commonly used YOLOv4, YOLOv5, YOLOv6, and YOLOv7 object-detection algorithms are selected. The vehicles and pedestrians in the images of high-concentration fog, medium-concentration fog, and low-concentration fog were, respectively, detected. Figure 29, Figure 30, Figure 31, Figure 32, Figure 33, Figure 34, Figure 35 and Figure 36 are shown below.

As can be seen from the visual target detection results of Figure 29, Figure 31, Figure 33 and Figure 35, which have never been defogging by the rapid defogging Optimization strategy 3 (the fusion of dark channel prior and Optimization strategy 3), YOLOv4, YOLOv5, YOLOv6 and YOLOv7 have different degrees of missed detection and false detection when detecting vehicles and pedestrians in images of high concentration fog, medium concentration fog and low concentration fog. By comparing the visual target detection results of Figure 30, Figure 32, Figure 34 and Figure 36 with the defogging optimization strategies 3 (the fusion of dark channel prior and Optimization Strategy 3), it can be seen that YOLOv4, YOLOv5, YOLOv6 and YOLOv7 have greatly improved the detection effect on vehicles and pedestrians in the defogging images with high concentration fog, medium concentration fog and low concentration fog. The visualized detection results further prove that the fast fog-removal Optimization Strategy 3 can detect vehicles and pedestrians in the foggy traffic environment.

## 4. Conclusions

This paper presents a fast fog-removal strategy suitable for a traffic environment. In the experiment, by reducing the size of the foggy picture to reduce the time of fog removal, the experiment formulated four optimization strategies. Four optimization strategies were combined with automatic color equalization (ACE), histogram equalization, and the dark channel prior algorithm, to compare the effectiveness of their optimization strategies. The experimental results show that the combination of Strategy 3 and the dark channel prior algorithm is the best optimization strategy. After bilinear interpolation, the original foggy picture is reduced by 256 times, and the dark channel prior algorithm is used for defogging. Then, the image after defogging is processed via 4-time up-sampling and Gaussian transform. The feature contour information of the image is more perfect, and the color information is also enhanced. The fast defogging strategy suitable for a traffic environment is compared with the original dark channel prior algorithm, to defog images of different concentrations, and the defogging time is reduced by 83.93–84.92%. The target-detection algorithm can successfully detect vehicles and lanes in high-concentration fog, medium-concentration fog, and low-concentration fog, and verify the effectiveness of the fast fog-removal optimization strategy.

The target detection algorithm can successfully detect vehicles and pedestrians in high-concentration fog, medium-concentration fog, and low-concentration fog, and verify the effectiveness of the fast fog-removal optimization strategy. The rapid defogging optimization strategy can show an excellent defogging performance in a daytime foggy environment, but the performance in a night foggy environment is very ordinary, which is what we need to work on in the future. In future work, we will further optimize the defogging strategy, to improve the defogging performance in a nighttime foggy environment.

## Figures and Tables

**Figure 3:**
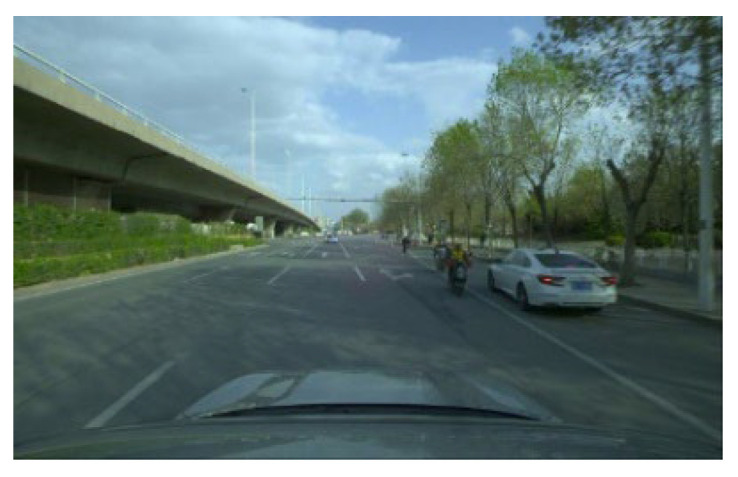
No-fog picture.

**Table 1:** Experimental environment configuration.

Name	Related Configuration
CPU	Intel Xeon Gold 6248R
GPU	RTX8000
GPU Accelerate library	CUDA 11.7, CUDNN 8.6.0
Deep learning framework	Pytorch 1.13.1
Operating system	Ubuntu 20.04

**Table 2:** A list of the 20 groups of experiments. Dark channel prior, Optimization Strategy 2, and Optimization Strategy 3 were used to calculate the minimum, maximum, and average defogging time of low-concentration fog pictures, medium-concentration fog pictures, and heavy-concentration fog pictures. The minimum values in the table are the minimum defogging times of low-concentration fog pictures, medium-concentration fog pictures, and heavy-concentration fog pictures in 20 groups of experiments, and the maximum values in the table are the maximum defogging times of low-concentration fog pictures, medium-concentration fog pictures, and heavy-concentration fog pictures in 20 groups of experiments. The average values in the table are the average defogging times of low-concentration fog pictures, medium-concentration fog pictures, and heavy-concentration fog pictures in 20 groups of experiments, respectively.

Algorithm	Low-Concentration-Picture Defogging Time (ms)			Medium-Concentration-Picture Defogging Time (ms)			Heavy-Concentration-Picture Defogging Time (ms)		
	Minimum	Maximum	Mean	Minimum	Maximum	Mean	Minimum	Maximum	Mean
Dark Channel Prior	52.95	58.23	54.61	52.26	56.61	53.40	52.07	56.55	54.35
Optimization Strategy 2	14.54	17.73	16.02	15.18	17.23	16.04	14.87	17.19	15.91
Optimization Strategy 3	8.4	8.78	8.59	8.4	8.94	8.58	8.35	8.73	8.53

**Table 3:** The DCP, DCPDN, AOD-NET, CAP, EN-DCP defogging algorithms, and our proposed algorithms provide the quantitative results of defogging for the low-concentration foggy images in Figure 2.

Evaluation Index	DCP	DCPDN	AOD-NET	CAP	EN-DCP	OUR
SSIM	0.7571	0.7751	0.7771	0.7673	0.7752	0.7774
PSNR	17.8324	16.9632	18.7632	17.8981	17.8651	19.1548
Average gradient	28.3689	28.3689	28.3689	28.3689	28.3689	28.3689
Defogging time (s)	0.0531	0.0402	0.0469	0.0108	0.0433	0.0084

**Table 4:** The DCP, DCPDN, AOD-NET, CAP, EN-DCP defogging algorithms and our proposed algorithms provide quantitative results of defogging for medium concentration foggy images in Figure 2.

Evaluation Index	DCP	DCPDN	AOD-NET	CAP	EN-DCP	OUR
SSIM	0.6987	0.6686	0.7139	0.6918	0.7023	0.7721
PSNR	17.9951	17.8921	18.2628	17.7897	15.6579	18.8902
Average gradient	18.5513	18.5513	18.5513	18.5513	18.5513	18.5513
Defogging time (s)	0.0530	0.0411	0.0471	0.0106	0.0435	0.0084

**Table 5:** The DCP, DCPDN, AOD-NET, CAP, EN-DCP defogging algorithms, and our proposed algorithms, provide the quantitative results of defogging for the heavy-concentration foggy images in Figure 2.

Evaluation Index	DCP	DCPDN	AOD-NET	CAP	EN-DCP	OUR
SSIM	0.7016	0.6879	0.7026	0.6899	0.6302	0.7042
PSNR	8.3707	8.298	9.0186	8.8167	8.3268	9.3659
Average gradient	6.2440	6.2440	6.2440	6.2440	6.2440	6.2440
Defogging time (s)	0.0532	0.0422	0.0480	0.0111	0.0443	0.0085

## Data Availability

The data are available in a publicly accessible repository. The data presented in this study are openly available in [repository name Berkeley DeepDrive] at (https://bdd-data.berkeley.edu/portal.html#download) (accessed on 1 May 2022), reference number [39].
